# Polycaprolactone/Amino-β-Cyclodextrin Inclusion Complex Prepared by an Electrospinning Technique

**DOI:** 10.3390/polym8110395

**Published:** 2016-11-18

**Authors:** Edgar Moyers-Montoya, Perla García-Casillas, Claudia Vargas-Requena, René Escobedo-González, Santos-Adriana Martel-Estrada, Carlos A. Martínez-Pérez

**Affiliations:** 1 Instituto de Ingeniería y Tecnología, Universidad Autónoma de Ciudad Juárez, Ave. Del Charro 450 Norte, Ciudad Juárez 32310, Mexico; edgar_moymon@hotmail.com (E.M.-M.); pegarcia@uacj.mx (P.G.-C.); 2 Instituto de Ciencias Biomédicas, Universidad Autónoma de Ciudad Juárez, Henry Dunant #4600, Ciudad Juárez 32310, Mexico; cvargas@uacj.mx; 3 Departamento de Ciencias Químicas, Universidad Autónoma de México, Campo 1, Av. 1 de Mayo S/N, Colonia Santa María las Torres, Cuautitlán Izcalli 54714, Mexico; renegerardo.escobedo@gmail.com; 4 Instituto de Diseño y Arte. Universidad Autónoma de Ciudad Juárez, Ave. Del Charro 450 Norte, Ciudad Juárez 32310, Mexico; mizul@yahoo.com

**Keywords:** electro-spinning, fibers, functional composites

## Abstract

Electrospun scaffolds of neat poly-ε-caprolactone (PCL), poly-ε-caprolactone/β-cyclodextrin inclusion complex (PCL/β-CD) and poly-ε-caprolactone amino derivative inclusion complex (PCL/β-CD-NH_2_) were prepared by the electrospinning technique. The obtained mats were analyzed by a theoretical model using the Hartree–Fock method with an STO-3G basis set, and characterized by X-ray diffraction (XRD), field emission scanning electron microscopy (FESEM), Fourier transform infrared spectroscopy-attenuated total reflectance (FTIR-ATR), differential scanning calorimetry (DSC), confocal-Raman spectroscopy, proton nuclear magnetic resonance (^1^HNMR) and contact angle measure (CA). Different mixtures of solvents, such as dimethylformamide (DMF)-tetrahydrofuran (THF), dichlormethane (DCM)-dimethyl sulfoxide (DMSO) and 2,2,2-Trifluoroethanol (TFE), were tested in the fiber preparation. The results indicate that electrospun nanofibers have a pseudorotaxane structure and when it was prepared using a 2,2,2-Trifluoroethanol (TFE) as solvent, the nanofibers were electrospun well and, with the other solvents, fibers present defects such as molten fibers and bead-like defects into the fiber structure. This work provides insights into the design of PCL/β-CD-NH_2_ based scaffolds that could have applications in the biomedical field.

## 1. Introduction

Electrospinning is a robust, easy and non-expensive method that has become very popular in recent years because of its versatility for spinning different kinds of polymers, natural and synthetic, with tunable properties such as highly superficial area, size and pore structure. Well-defined nano and microfibers can be produced by varying voltage, work distance, density and polymer concentration, and solvent types, among others. The electrospinning technique has been used to produce fiber materials for diverse applications from electronics to biomedical applications studied [1,2]. On the other hand, polycaprolactone (PCL) is an aliphatic, semi-crystalline polyester polymer that has been approved by the American Food and Drug Administration (FDA) for biomedical applications. Besides the good biocompatibility, PCL have properties like good solubility in many kinds of organic solvents, low melting point close to 60 °C, and remarkable blend-compatibility that make it an excellent candidate for research in tissue engineering [3]. Although PCL is biocompatible and a material that is very easy to handle and shape, its use in biological applications is limited by its hydrophobicity and lack of active sites. These would allow it to immobilize or to attach biomolecules that could interact positively with cells to enhance its properties for tissue engineering and drug delivery. Some of the applied strategies have included surface and polymer chemical modification, copolymerization of two or more polymers with incorporated nanoparticles, development of bicomponent fibers by coaxial electrospinning, as well as post-treatments and coating nanofibers with bioactive materials [4,5]. However, some of these strategies may compromise the physicochemical properties of PCL [6]. Therefore, it is necessary to develop new methods to overcome these limitations. One alternative to adding bioactive sites to PCL can be the preparation of the non-covalent inclusion complex (IC) with cyclodextrins (CD), which are cyclic molecules composed of six, seven or eight glucose units (α, β or γ) with an internal cavity of high hydrophobicity [7,8,9,10]. CD allows the formation of complexes with other molecules such as polymers. Previous investigations have shown that IC can be obtained with polymers and any kind of CD; however, it was found that α-CD provides better performance and higher yield to form IC with PCL than the other CDs. Despite this, β-CD has been widely exploited for the preparation of drug delivery systems because it possesses an effective performance yield in several cases [11,12]. The β-CD is widely used for drug delivery systems; however, its lower solubility, in comparison with α and γ-CD, make the preparation of inclusion complexes with PCL polymer more complicated [13]. However, the inconvenience of its low solubility has been overcome by attaching amino groups (–NH_2_) on the external face of the CD, which increase the polarity and improve the solubility in aqueous medium, and the same group has the functionality to adhere molecules with biological interest [14].

Additionally, the preparation of electrospun Polycaprolactone with α and γ-cyclodextrin composite fibers, and the effect of these cyclodextrins on the thermal and crystal nucleation behavior was studied by Tonelli et al. [15]. The results showed nanofibers with an average diameter of 400 nm increasing with the amount of cyclodextrins, and the PCL/CD composites exhibited higher crystallization temperatures and sharper crystallization exotherms with increased CD loading, representing the capability of CDs to nucleate PCL crystallization. The nanofibres exhibited an increasing of the hydrophilicity and potential use in medical applications. Moreover, in 2015, this same research group reported the electrospun obtaining of the PCL and β-cyclodextrin composite nanofibers having diameters of around 500 nm. The outcomes suggest tailoring polymer-CD nanostructures for specific applications in wound odor absorbance, surface grafting of chemical moieties, and vehicles for drug delivery, for example [16]. Regarding the synthesis of inclusion complex nanofibers by electrospining, previous work reported the production of non-stoichiometric PCL and an α-cyclodextrin inclusion complex of electrospun nanofibers, a bead free material with increases in melting and the crystallization temperatures. Furthermore, the mechanical properties of the composite webs, with the addition of the ICs, increased the tensile modulus and ultimate tensile strength of the composite fibers with reduction of their extensions at the break point [17]. More recently, multifunctional scaffolds containing neat poly(ε-caprolactone) (PCL) and α-cyclodextrin pseudorotaxanated in α-cyclodextrin form were fabricated by electrospinning method using chloroform (CFM) and the mixture CFM/dimethylformamide (DMF) as solvents. The obtained material from CFM showed differences in the morphology and the arrangement of the chains, also exhibiting higher moduli and lower elongations at break compared to neat PCL nanowebs and PCL/α-CD nanowebs electrospun [18]. For these reasons, in this work, a β-CD amine derivative is used, prepared on the basis of the synthesis of 6-*O*-monotosyl-6-desoxy-β-CD (this first precursor allows the synthesis of mono-substituted CD derivative with attached functional groups to the external face of the CD) [19] and subsequently modified to obtain the amino derivative: 6-Monodeoxy-6-monoamino-β-cyclodextrin (β-CD-NH_2_) [9]. Although a complex between PCL and α-CD has already been reported [14], in this work, electrospun nanofibers of PCL and the β-CD-NH_2_ complex is reported for the first time, which introduces a high level of performance and new perspectives for the use of β-CD in drug delivery and tissue engineering by scaffolds’ nanofiber systems—particularly, the β-CD-NH_2_ that will increase the surface bioactivity, providing the possibility of anchor molecules of biological interest on the material surface without the need of modifying the PCL structure.

## 2. Materials and Methods

Poly(ε-caprolactone) PCL with a MW of 70,000–90,000 Da, β-Cyclodextrin reagent grade and CM Sephadex C-25 were acquired from Sigma-Aldrich (St. Louis, MO, USA); Acetone, 2,2,2-Trifluoroethanol (TFE), dimethylformamide (DMF) dichlormethane (DCM) and dimethyl sulfoxide (DMSO). All of them were purchased from JT Baker (Ecatepec, México State, México), and used without further purification. The cyclodextrin prior its use was dried in an oven vacuum at 80 °C for 24 h.

### 2.1. Synthesis of 6-Monodeoxy-6-monoamino-β-cyclodextrin (β-CD-NH_2_)

The β-CD-NH_2_ was synthesized by the substitution of one amine group located on the outside face of the β-CD by 6-*O*-monotosyl and was purified by ionic exchange chromatography using a Sephadex C-25 matrix [9]. The compound was characterized by Fourier transform infrared spectroscopy-attenuated total reflectance (FTIR-ATR), differential scanning calorimetry (DSC), proton nuclear magnetic resonance (^1^HNMR) spectroscopy and Fast atoms bombardment mass MS-FAB+ spectrometry. The product purity was determined by measuring its melting point by Differential Scanning Calorimeter DSC (using a SDQ 600, TA instrument, New Castle, DE, USA), its molecular weight was obtained by FAB mass spectrometry (JEOL JMS-700, Tokyo, Japan). The chemical structure was determined by means of proton nuclear magnetic resonance at 300 MHz in DMSO-_d6_ H^1^NMR (Varian Mercury-300, Palo Alto, CA, USA).

### 2.2. Preparation of Inclusion Complex of PCL with β-CD and 6-Monodeoxy-6-monoamino-β-cyclodextrin (β-CD-NH_2_)

An inclusion complex of PCL with β-CD and β-CD-NH_2_ was prepared following the procedure published by Yoshinori Kawaguchi et al. [12] and Jiahan Zhan et al. [14]. In addition, 1.0 g of PCL (Mw 70,000–90,000 Da; Sigma-Aldrich, St. Louis, MO, USA) was dissolved in 60 mL of acetone and heated at 50 °C in an oil bath. Then, 0.5 g of β-CD were dissolved in 10 mL of DMF and then added dropwise to the PCL solution. After stirring for 2 h, the mixture was left to cool down to room temperature and slowly stirred overnight in order to evaporate the acetone. The obtained material was washed several times with distillated water to remove any unthreaded CD. Finally, the material was dried in an oven at 37 °C and under reduced pressure of −25 in Hg. The obtained PCL/CD IC was characterized by Fourier transform infrared FT-IR (Nicolet 6700 Thermo Scientific, Waltham, MA, USA), confocal Raman microscopy (Alpha 300R Witec Control, Witec focus innovation, Ulm, Germany), differential scanning calorimetric (DSC 200 NETZSCH, Netzsch-Gerätebau GmbH, Selb, Germany) 25–300 °C, 5 K/min, and proton nuclear magnetic resonance at 600 MHz on DMSO-_d6_.

### 2.3. Theoretical Study

The geometries of the arrangements of the investigated complex, β-CD and β-CD-NH_2_ were optimized with the GAUSSIAN 09 program (Gaussian Inc., Wallingford, CT, USA) [20], employing the Hartree–Fock method, with STO-3G basis set. All of the parameters were relaxed and all calculations converged to structures of maximum stability, which corresponds to an energy minimum [21,22,23].

### 2.4. Electrospinning

The PCL and IC solutions were prepared with dissolving in DMF/THF [24], DCM/DMSO [14] and TFE [25] as reported in previous work. The solutions’ electrospinning was made searching the best mats varying the polymer/solvent ratio of 3% to 12% (*w*/*v*); the best morphology was selected considering the smaller diameter nanofibers without beading defect. The viscosity profile and molecular weight was measured by rheological analysis (TA 2000Ex AR, TA instruments, New Castle, DE, USA) [26]. The solution was drawn into a 10 mL glass syringe with a 30 G needle, optimizing the electrospinning from 8 to 14 kV (Gamma High Voltage Research, Ormond beach, FL, USA) and 0.8–1.2 mL/h (KDScientific 200, Holliston, MA, USA) and 2000 rpm on a cylindrical collector. The IC nanofiber was characterized by SEM (JEOL JSM 7000F, JEOL, Tokyo, Japan), confocal Raman microscopy, thermogravimetric analysis (Witec focus innovation, Ulm, Germany) and DSC. The wettability was estimated by contact angle and spreading coefficient (DSA30 Krüss, Matthews, NC, USA).

## 3. Results

### 3.1. Synthesis of 6-Deoxy-6-amine-β-cyclodextrin

The first stage of this work was the synthesis of the β-cyclodextrin amine derivative with a final yield after its purification of 0.4, which is in accordance with other works [27,28,29,30]. The product was characterized by spectroscopic methods like FT-IR, mass spectrometry and ^1^HNMR. The infrared spectra showed the following bands 3350, 1640, 1550 cm^−1^, and these are attributed to N–H vibrational bands from primary amines. Additionally, the characteristic bands of the cyclodextrin moiety (3300, 2900, 1180–1100) were found. The characterization of the amine cyclodextrin was achieved using FAB+-MS. In this analysis, the sample was dispersed in thioglicerol matrix and bombarded with accelerated xenon ions. This process typically gives two types of “molecular” ions, which include molecular ions of the target compound plus hydrogen or sodium atoms (those atoms were present in the matrix). The β-CD-NH_2_ spectrum shows the peaks of 1157, which correspond to the molecular ion of this amine derivative plus the sodium atom; a typical pattern in this ionization method and signaling the monosustitution of the β-Cyclodextrin [31,32]. Finally, the signals presented in ^1^HNMR were the same as previously reported. 6-deoxy-6-amine-beta-cyclodextrin (β-CD-NH_2_); mp 201 °C. IR-ATR (cm^−1^): 3350, (N–H), 3300 (–OH), 2900 (C–H), 1640 (N–H), 1550 (N–H) 1180–1100 (C–O–C). MS-FAB+ (*m*/*z*): 1157 [M + 23], 1179 [M + 45], M^+^ not observed. ^1^HNMR (300 MHz, DMSO-_d6_, δ ppm): 5.81–5.6 (m, 14H), 4.9–4.80 (m, b, 6H), 4.6–4.4 (m, b, 6H) 3.65–3.50 (m, b, 28H), 3.42–3.33 (overlap with H_2_O, m, 16H).

### 3.2. Inclusion Complexes β-CD and β-CD-NH_2_ with PCL and Electrospinning

**Figure 1:**
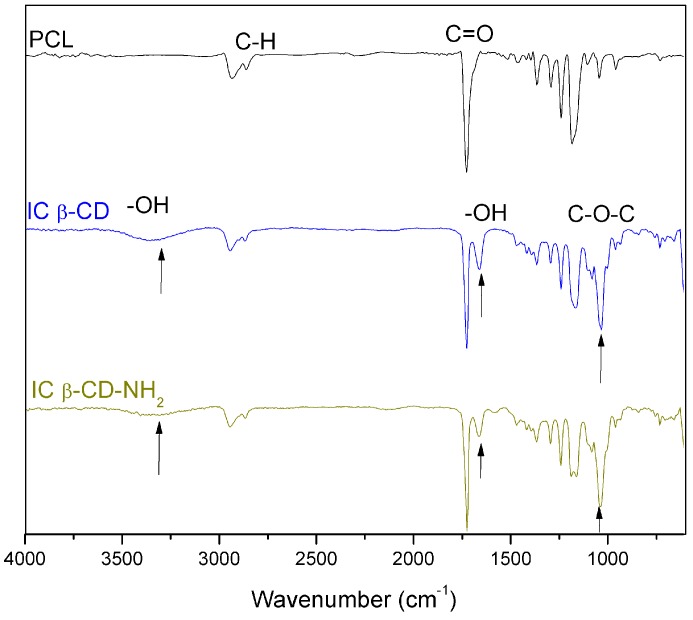
FTIR-ATR (Fourier transform infrared spectroscopy-attenuated total reflectance) spectra of PCL (poly-ε-caprolactone), β-CD (β-cyclodextrin) and β-CD-NH_2_ IC (inclusion complex) nanofibers.

The electrospun nanofibers of inclusion complexes obtained among β-CD or β-CD-NH_2_ and the PCL were characterized by IR-ATR and compared with PCL nanofibers, as can be seen in Figure 1. Both inclusion complexes’ fibers presented a broad band at 3400 cm^−1^. Furthermore, three bands corresponding to C–O–C bending mode of the IC were observed around 1150, 1080 and 1030 cm^−1^. The symmetrical and asymmetrical stretching from the bonds C-H of the PCL aliphatic chains were located at 2850 and 2930 cm^−1^. Additionally, a prominent and characteristic band of the ester carbonyl group C=O was presented at 1732 cm^−^^1^.

The spectrum of both inclusion complexes presented a slight shift in the C=O band of the PCL and exhibited a new small band at ~1650 cm^−1^. According to Shin K-M., et al. [33,34], this band is indicative of the formation of the inclusion complex. These changes are the result of the resolution of the carbonyl bands of crystalline and amorphous PCL by the change in the crystallinity degree of PCL due to the formation of inclusion complex. Moreover, there is the presence of intramolecular hydrogen bonds among the carbonyl group of PCL and the hydrogen of the cyclodextrin [33]. Moreover, the hydroxyl bands for the CDs are shifted to high values in the IC spectra, and this phenomenon is attributed also to the formation of hydrogen bonds between the carbonyl groups of PCL and hydroxyl groups of the CD [33,34].

**Figure 2:**
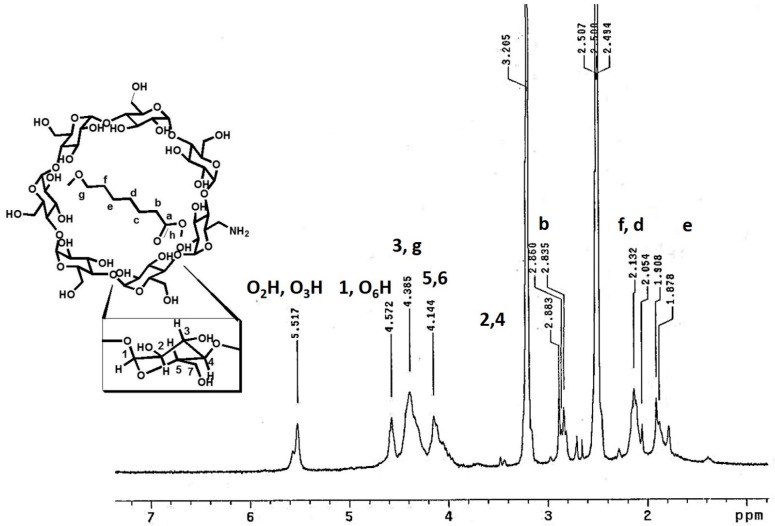
The ^1^H NMR (Proton Nuclear Magnetic Resonance) spectrum of β-CD-NH_2_ IC (300 MHz, DMSO-_d6_/Acetic acid-d4).

The inclusion complex of the PCL with β-CD-NH_2_ was characterized by H^1^NMR and the spectrum is shown in Figure 2. The aliphatic hydrogens (e, d, and f) of PCL were found at 1.8 and 2.1 ppm. In this sense, the hydrogens in alpha position to carbonyl groups appeared at 2.8 ppm. In addition, the hydrogens in the oxygen base of carboxyl group were presented at 2.8 ppm [12,35]. The hydrogen 2,4 of the cyclodextrin are overlapping in the signal of 3.2 ppm. The multiple signals between 4.0–4.2 ppm; 4.2–4.45 ppm and 4.5–4.6 ppm correspond to the protons 5, 6, 3 and the hydrogen 1 and the hydroxyl group O_6_H of β-CD-NH_2_, respectively. Finally, the signals presented around 5.6 ppm correspond to the hydroxyl groups O_2_H and O_3_H [36,37,38]. Furthermore, the chemical shift of the host and guest suffer a change, compared with the free molecules, as a result of the IC formation (vide supra).

**Figure 3:**
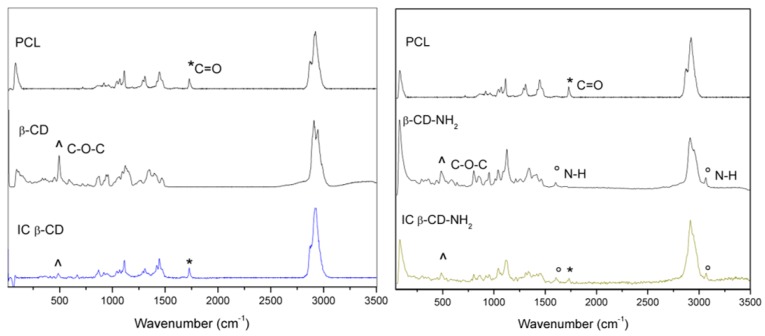
Raman spectra for PCL, IC nanofibers and the cyclodextrin precursors: (∧) indicate characteristic bands of the cyclodetrins, (*) characteristic bands of PCL and (°) characteristic band of amine group.

In Figure 3, the Confocal Raman characterization for the PCL, PCL/β-CD and PCL/β-CD-NH_2_ fibers are shown. The IC’s spectra show typical absorptions bands observed at 500–1450 cm^−1^, corresponding to the cyclodextrin compounds. The signals present at 1700 cm^−1^ and around 3000 cm^−1^ are attributed to the carbonyl group and to the polyester chain of poly ε-caprolactone vibration [39,40]. Furthermore, the shift in the bands is due to the formation of the IC, which is indicative of the guest–host interaction [39]. Moreover, the β-CD-NH_2_ IC presents an additional stretching band around 3100 cm^−1^ and other bending bands around 1600 cm^−1^ that are associated with the vibrations of the N–H bond [40].

**Figure 4:**
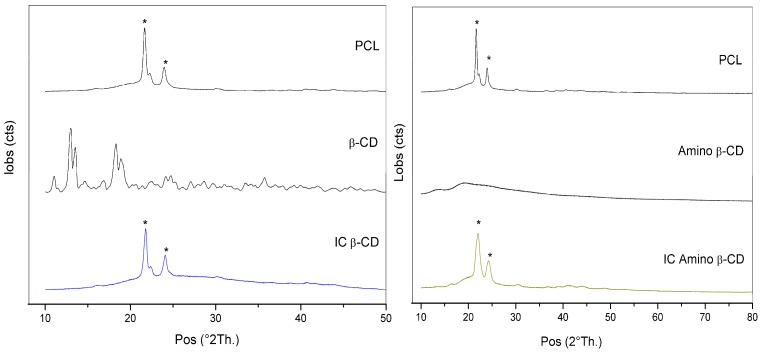
XRD (X-ray Diffraction) pattern for PCL, β-CD IC and β-CD-NH_2_ IC fibers: the asterisks designate a characteristic peaks of PCL.

The arrangement of the inclusion complex was studied by powder X-ray Diffraction (Figure 4). Previous works reported, for the pure PCL, the presence of characteristic peaks around 2θ = 22° to 24°, corresponding to reflections’ planes (110) and (200). However, when the polymer forms ICs, its cage arrangement reflections disappear and a peak at 2θ = 20° appears, attributed to columnar inclusion complex formation [14,15,18,33]. Regarding the diffraction patterns, the results of PCL/β-CD inclusion complex (CD IC) showed the two peaks at 21° and 24° (2θ), which correspond to the orthorhombic planes PCL crystalline regions in the material [14,33].

Despite IC being prepared with α-CD, the presence of a peak at 20° (2θ) has been reported, suggesting the influence of α-CD on the crystalline moiety of the polymer, while β-CD does not [14]. The absence of the 20° peak can be attributed to the distribution of the cyclodextrin in the polymer chain but not significantly in the crystalline phase of the PCL [41]. On the other hand, the diffraction pattern of the β-CD-NH_2_ IC displayed a weaker and broader peak around 20° (19.3°) compared with β-CD IC, which is attributed to the presence of some PCL chains threading through the cyclodextrin cavity as a consequence of the greatest hydrogen bonding in the amine derivative complex in contrast with the unmodified cyclodextrin [37]. The weaker intensity and the broader form of this emerging peak (19.3°) is a consequence of the stoichiometry of the IC. The molar ratio of cyclodextrin-PCL is less than 1:1, which provokes the increase of the reflection peak intensity (110), as a consequence of chains not included with the decrease of the reflection peak intensity of the complex [17].

The melting points (T*m)* of the materials were 57.9, 66.1 and 58.2 °C for PCL, β-CD IC and β-CD-NH_2_ IC, respectively. The variation in the melting point is an indication of the formation of the inclusion complex between the PCL and the CD, the change being dependent on the amount of the cyclodextrin covering the chains of PCL [17]. The results showed an increase in the melting point when the cyclodextrins were present in the material. In this arrangement, two neighboring CDs (head to head or tail to head) are stacked to form an endless column, and the polymer chains are included in the narrow channels that run continuously down each CD column. The major driving forces leading to the formation and stabilization of the IC are the hydrophobic interaction between the CD cavity and the polymer chains. Some works have described the interaction between the PCL and CDs, such as the channel type. In these works as well in the present research, there is evidence of the hydrogen bond between the carbonyl group of PCL and the hydroxyl groups of the CD IC [14,33,34].

### 3.3. Theoretical Study

**Figure 5:**
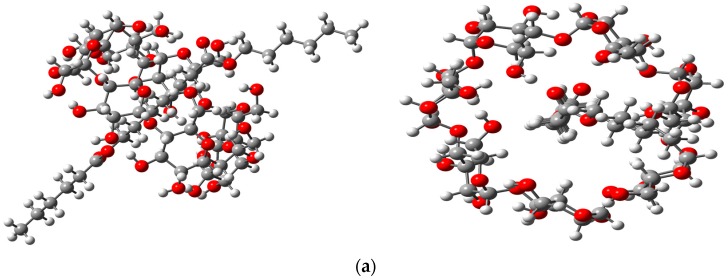

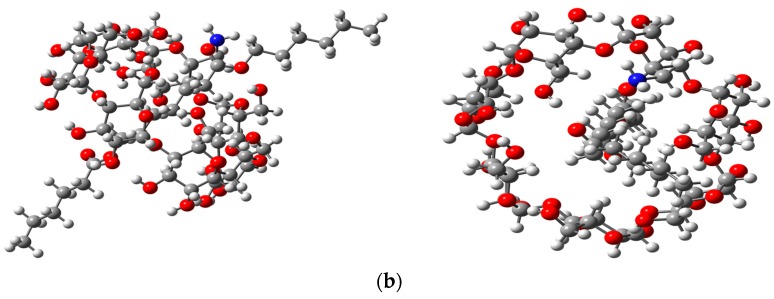
HF(Hartree-Fock)/STO-3G optimized geometries of the IC stoichiometry 1:1: (**a**) PCL/β-CD IC and (**b**) PCL/β-CD-NH_2_ IC. Left images correspond to the side view and right images correspond to frontal view.

In order to get a better understanding of the interactions between the β-CD or β-CDNH_2_ with PCL, a dimeric chain of PCL as a model, was studied by a theoretical method. The more stable structures that were obtained are shown in Figure 5. The optimized structures showed the aliphatic chain inside of the cyclodextrins’ hydrophobic cavity. Additionally, one of the carbonyl groups of PCL is close to the hydroxyl groups on the minor face of the β-CD and β-CD-NH_2_, suggesting the presence of hydrogen bonds between them and the channel type structure. Moreover, supporting the idea of the β-CD-NH_2_, IC follows the same type of interaction as β-CD. These results are in agreement with the characterization of the compounds and others reported with α-cyclodextrin. Furthermore, the interaction energy of the PCL with β-CD and β-CD-NH_2_ was calculated at the same theory level. In the case of unmodified β-cyclodextrin, the interaction energy in the gas phase was −12.986 Kcal/mol in contrast with −13.140 Kcal/mol for the amino derivatives. The difference of the interaction energies denotes more stability of the inclusion complex with the β-CD-NH_2_. In the same way, the optimized structures were used to obtain the theoretically IR and Raman spectra of both complexes. These results also infer the formation of pseudorotaxane fibers structures being in agreement with the experimental data [21].

**Figure 6:**
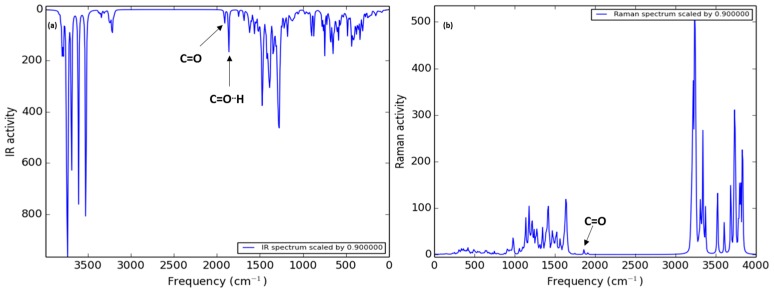
HF/STO-3G calculated Infrared (IR) and Raman spectra of PCL/ β-CD IC.

**Figure 7:**
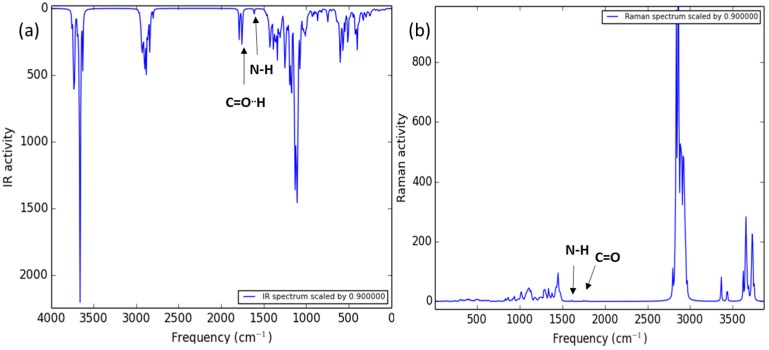
HF/STO-3G calculated IR (**a**) and Raman (**b**) spectra of PCL/β-CD-NH_2_ IC.

The theoretical IR spectra of both ICs (Figure 6 and Figure 7) showed the bands of the hydroxyl groups at high frequency (4500–3500 cm^−1^) and the presence of two vicinal bands (2100 to 2000 cm^−1^). These bands are attributed to carbonyl groups of PCL, where the second band at low frequency refers to the carbonyl group with a hydrogen bond with the minor face to the CD structure, in agreement with the experimental IR spectra. In addition, the presence of the characteristic band corresponds to the C–O–C bound vibration (around 1400 cm^−1^). In the case of the theoretical Raman spectra, it is possible see the same pattern of bands as the experimental spectra. In the case of the β-CD-NH_2_ IC, the characteristic band of the amine group is shown in the experimental and theoretically spectra (2006 cm^−1^). Furthermore, the presence of the carbonyl band of PCL is observed at 2064 cm^−1^. These results support the arrangement of the β-CD-NH_2_ with the PCL proposed and signaled good results using this theory level in the calculations. Finally, the β-CD-NH_2_ IC also presented the vibrational band of the N-H bond (around 1789 cm^−1^).

In the case of the theoretical Raman spectra, it is possible to see the same pattern of bands as the experimental spectra. In the case of the β-CD-NH_2_ IC, the band characteristic of the amine group shown in the experimental spectra is theoretically present (1789 cm^−1^). Furthermore, the presence of the carbonyl band of PCL is observed at 1947 and 1984 cm^−1^. These results support the arrangement of the β-CD-NH_2_ with the PCL proposed and signaled good results using this theory level in the calculations.

### 3.4. Electrospinning

**Figure 8:**
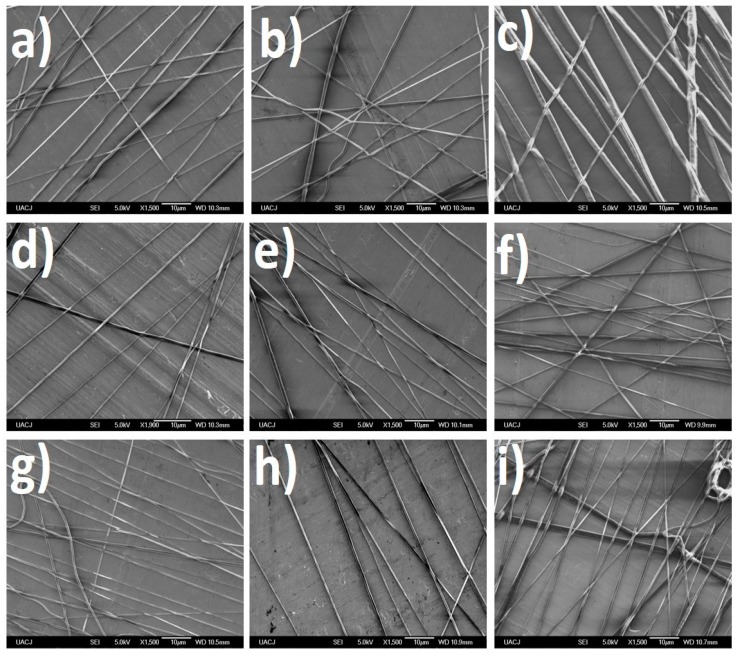

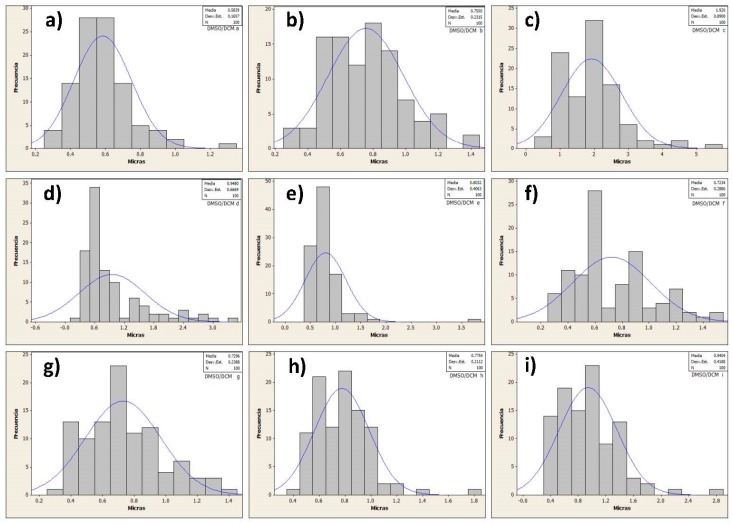
Optimization process of PCL (12%) electrospun in DCM/DMSO (dichlormethane/dimethyl sulfoxide): (**a**) 10 kV, 0.7 mL/h, 18 cm; (**b**) 10 kV, 0.7 mL/h, 14 cm; (**c**) 10 kV, 0.7 mL/h, 10 cm; (**d**) 10 kV, 0.5 mL/h, 14 cm; (**e**) 10 kV, 1.2 mL/h, 14 cm; (**f**) 10 kV, 2 mL/h, 14 cm; (**g**) 12 kV, 1.2 mL/h, 14 cm; (**h**) 14 kV, 1.2 mL/h, 14 cm; (**i**) 16 kV, 1.2 mL/h, 14 cm. SEM images (**top**) and size distribution (**bottom**).

The electrospinning optimization process was developed in first stage for the DMSO/DCM mixture, evaluating the effect of the process parameters on the fiber size. The SEM images (Figure 8, top) showed the nanofiber production with average diameter around 750 nm for DCM/DMSO and a wide range of dispersion. The size of the fibers obtained was the bigger of the systems tested. Moreover, it was discarded because the high evaporation temperature of this solvent caused the melting of nanofibers.

**Figure 9:**
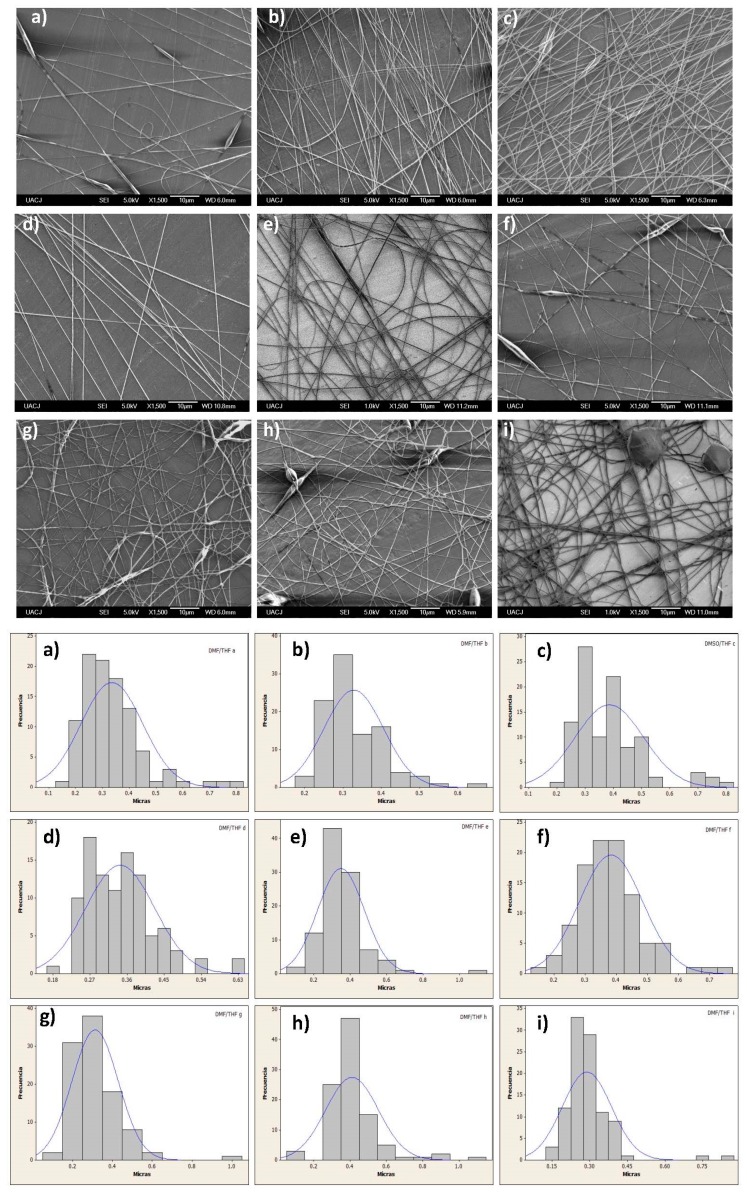
Optimization process of PCL (12%) electrospun in DMF:THF (dimethylformamide:tetrahydrofuran) (**a**) 10 kV, 0.7 mL/h, 18 cm; (**b**) 10 kV, 0.7 mL/h, 14 cm; (**c**) 10 kV, 0.7 mL/h, 10 cm; (**d**) 10 kV, 0.5 mL/h, 14 cm; (**e**) 10 kV, 1.2 mL/h, 14 cm; (**f**) 10 kV, 2 mL/h, 14 cm; (**g**) 12 kV, 1.2 mL/h, 14 cm; (**h**) 14 kV, 1.2 mL/h, 14 cm; and (**i**) 16 kV, 1.2 mL/h, 14 cm. SEM images on the (**top**) and size distribution on the (**bottom**).

Regarding the second system, the SEM images (Figure 9, top) of the nanofibers provided evidence of the presence of smaller nanofibers, diameters around 300 nm; however, they were discarded because they always produced bead defects in the nanofibers.

**Figure 10:**
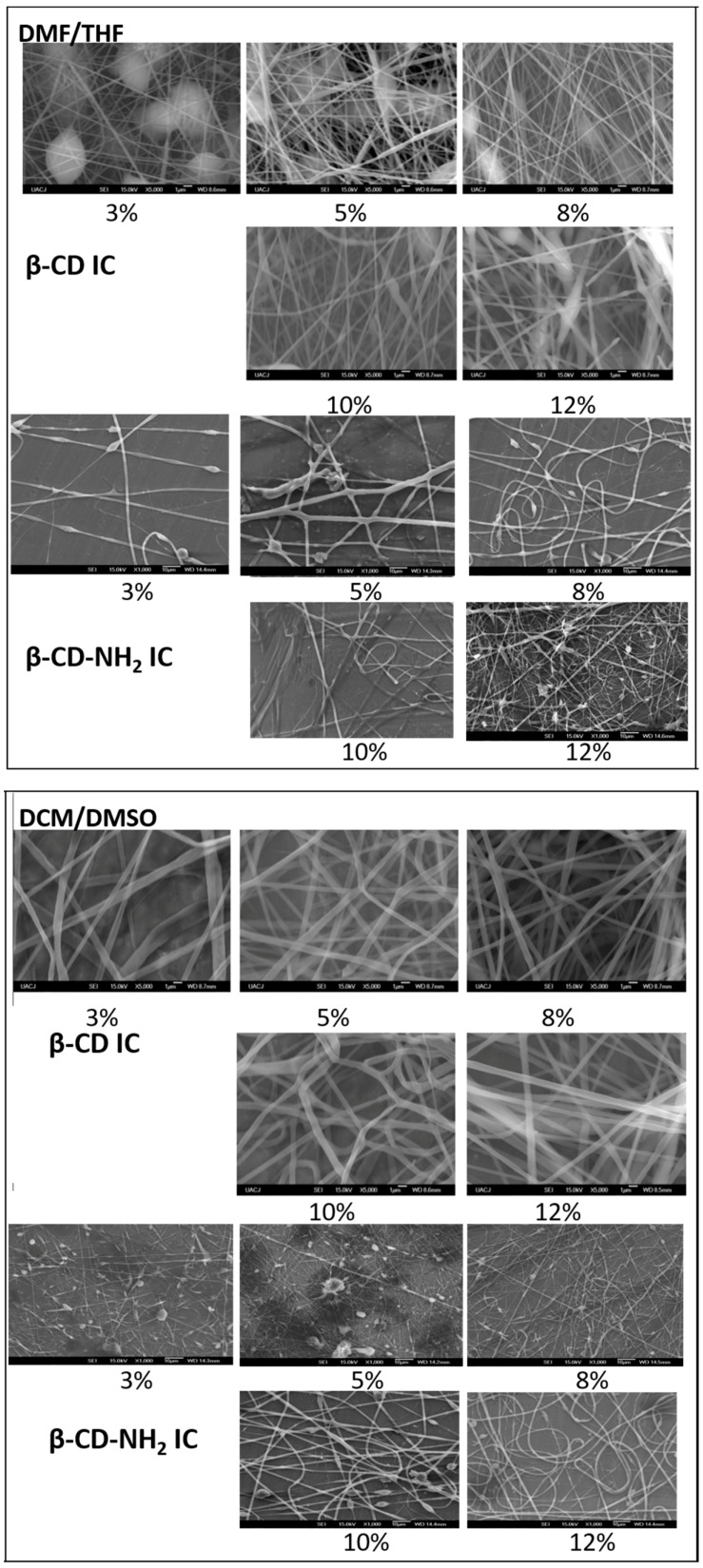
Electrospinning optimization process, with different concentrations of β-CD/PCL and β-CD-NH_2_/PCL using two solvent mixes: DMF/THF (**top**) and DCM/DMSO (**bottom**).

The electrospinning parameters where the PCL fibers had the best morphology in the optimization process were tested from 3% to 12% (polymer/solvent ratio) with β-CD and β-CD-NH_2_ ICs (Figure 10). The results showed the same behavior as that the PCL nanofibers. The fibers obtained with the DMSO/DCM had fiber diameters of around 1 µm due to the melting of the nanofibers. Inspecting the fibers produced with the system DMF/THF revealed the presence of beads in all of the concentrations of β-CD/PCL and the experimental conditions. These results were in agreement with those observed previously in the PCL material, permitting the discarding of this solvent mix as a system of the electrospinning.

Considering the previous results, the electrospinning process was tested, used as solvent 2,2,2-trifluroethanol in an attempt to improve the morphology of the nanofibers. The best parameters for the smaller fibers were 8 kV and 0.8 mL/h, employing a collector distance of 18 cm and a rate of 2000 rpm.

**Figure 11:**
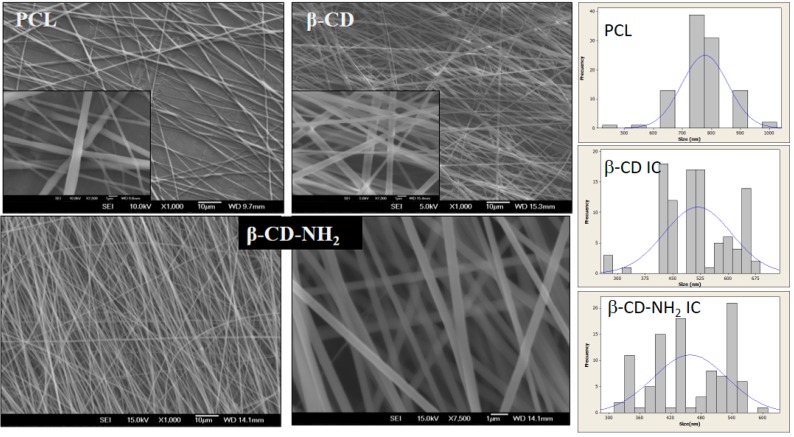
SEM Images of PCL and IC nanofibers scaffolds from 12 wt % IC /TFE (2,2,2-trifluoroethanol), 0.8 mL/h, 8 kV, 18 cm and 2000 rpm of the collector rotation rate. PCL and β-CD IC nanofibers; β-CD-NH_2_ IC nanofibers (1000× and 7500×) obtained in the same conditions.

The morphology of the samples showed in all concentrations homogenous fibers without beads or melting effects, and diameters in the range of 400 to 800 nm. The smaller fibers’ sizes were obtained with 12% of the material (PCL, β-CD/PCL and β-CDNH_2_/PCL) in the TFE. Representative SEM images of the electrospun materials at 12% are shown in Figure 11. Well-defined nanofibers of PCL can be appreciated, as well for the PCL/β-CD IC and PCL/β-CD-NH_2_. The produced neat PCL nanofibers had diameters of 520 ± 150 nm. Meanwhile, for the PCL/β-CD IC, the average diameter was 719 ± 168 and 462 ± 77 nm for the PCL/β-CD-NH_2_ IC.

**Figure 12:**
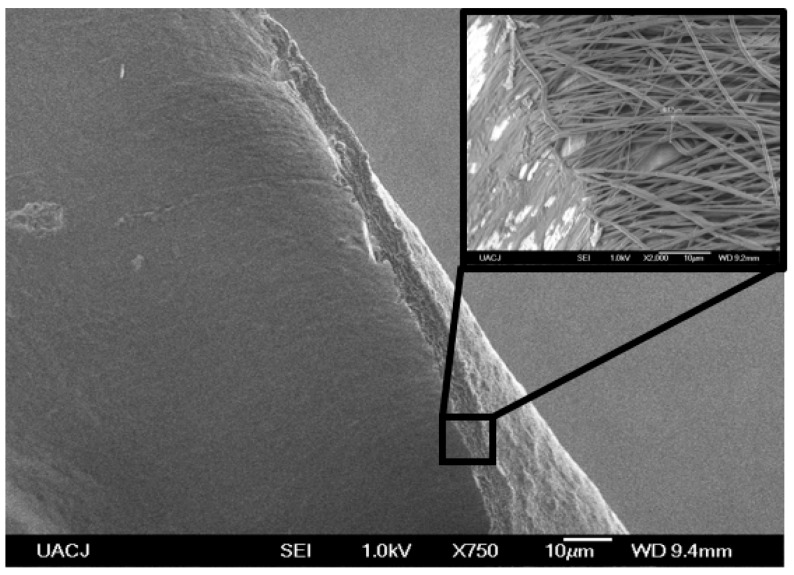
SEM image (750×) of the side perspective of a film obtained with the β-CD-NH_2_ /PCL in the TFE electrospinning process (inset 2000×).

The images show a reduction in diameter size and dispersion of the PCL/β-CD-NH_2_ IC fibers as a result of a better solubility provided by the electrons of the amino groups [9,26]. In each case, a film with a size of 10 × 20 cm, a thickness of around ~6 μm, and a weight of 288 mg was obtained in 3 h of electrospinning, as is shown in Figure 12.

The viscosity of polymer solutions (12%) in TFE at 25 °C for PCL, β-CD IC and β-CD-NH_2_ were observed to be 802.12 ± 37.56, 817.46 ± 89.78, and 691.35 ± 44.9 mPa S, respectively. The increment in the viscosity of the jet would facilitate the formation of a solid skin on the jet surface, and the polymer chains would face difficulties in their migration from the surface to the liquid core with the consequence of irregular flow that produces a higher dispersion of diameters on electrospun nanofibers [1,26]. In addition, PCL/β-CD-NH_2_ solutions produce nanofibers with less variability and with a narrow dispersion, having lower viscosity at the same concentrations and temperature [25,42].

**Figure 13:**
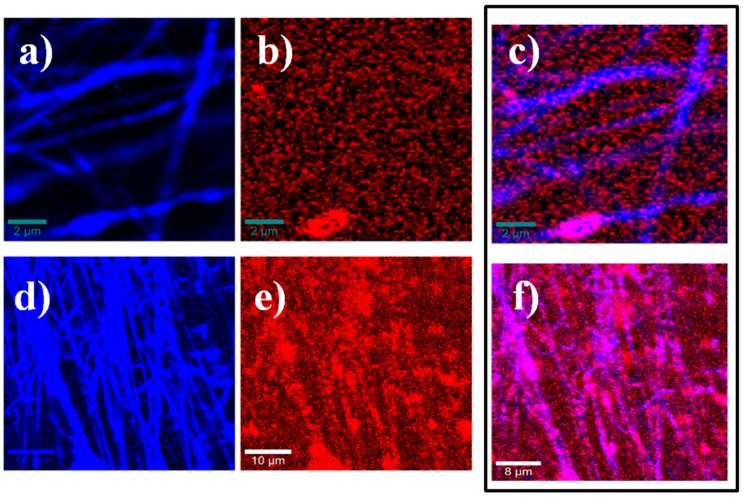
Raman images of electrospun IC nanofibers: PCL/β-CD (**a**–**c**); β-CD-NH_2_ IC nanofibers (**d**–**f**). The multivariate data analysis algorithms for HCA (hierarchical cluster analysis) were used to discriminate between the signals and represent the distribution in the IC fibers. In **blue**, the image by integration for the peak at ~1700 cm^−1^ is attributed to the C–H stretching vibrations in PCL; in **red**, the integration for the peak at ~500 cm^−1^ is attributed to the different cyclodextrins. Finally, the images (**c**,**f**) show the combined images for each IC, showing in **purple** the distribution of cyclodextrin on the PCL.

The Confocal Raman image analysis (Figure 13) shows the complex in the electrospun nanofibers, comparing the image generated by the integration of the Raman signal appearing at ~1700 cm^−1^ (blue) corresponding to C–H stretching vibrations in PCL, and the image generated by the signal at ~500 cm^−1^ (red) corresponding to C–O–C (ether group) of cyclodextrins [43]. In each case, the ciclodextrin distribution on the PCL can be observed in the third image (purple). A better performance can be observed on complexation with β-CD-NH_2_ than that with β-CD, and there is a consistent presence of β-CD-NH_2_ and a better distribution on the fiber.

**Figure 14:**
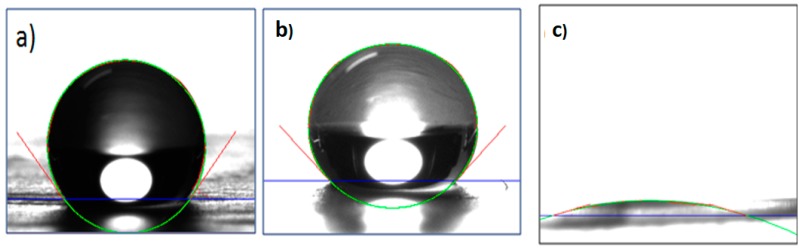
Water Contact Angle (CA) for nanofibers of: (**a**) PCL; (**b**) β-CD IC; and (**c**) β-CD-NH_2_IC.

Wettability is another important parameter for biomedical applications, although there are several parameters that must be carefully taken into consideration in the design and fabrication for materials that will be in contact with biological systems. Wettability could be the first sign for predicting biocompatibility of non-biological materials because, in the first contact with biological systems, a protein layer formation is the first step and first indication of the possible compatibility of the materials [44]. In Figure 14, the wettability measured trough the contact angle for PCL, PCL/β-CD and PCL/β-CD-NH_2_ IC is shown. According to water contact angle measures for neat PCL and PCL/β-CD that were 135.4° ± 2.18° and 124.5° ± 0.44°, respectively, these mats have a very hydrophobic surface, which will not favor the formation of the protein layer. On the other hand, the electropsun PCL with β-CD-NH_2_ presents high hydrophilicity, with a contact angle of 1.3° ± 0.61°. Its high hydrophilicity is attributed to the amino groups that lead to an almost total water dispersion on the material. This high hydrophilicity is attributed to the amino groups that promote solubility and water dispersion.

## 4. Conclusions

Neat PCL, PCL/β-cyclodextrin and PCL/β-cyclodextrin-NH_2_ inclusion complexes were successfully prepared by an electrospinning technique. By tuning the parameter process of these materials, well-defined nanofibers with a narrow distribution can be fabricated. We report for the first time the fabrication of PCL/β-CD-NH_2_ where a pseudorotaxane structure was obtained according to both the theoretical and characterization results. The solvent plays a crucial role, and, in this system, TFE proved to be the most effective solvent for the preparation of the material nanofibers, unlike the other solvents where no fibers were formed or fibers with bead-like defects were produced. The novel PCL/β-CD-NH_2_ pseudorotaxenes are functional composites that could be suitable for different applications, especially in biomedical and drug delivery systems.
